# Novel early-onset Alzheimer-associated genes influence risk through dysregulation of glutamate, immune activation, and intracell signaling pathways

**DOI:** 10.21203/rs.3.rs-4480585/v1

**Published:** 2024-06-05

**Authors:** Carlos Cruchaga, Joseph Bradley, Daniel Western, Ciyang Wang, Eder Lucio Da Fonseca, Achal Neupane, Jiji Kurup, NIcholas Ray, Melissa Jean-Francois, Priyanka Gorijala, Kristy Bergmann, John Budde, Eden Martin, Margaret Pericak-Vance, Michael Cuccaro, Brian Kunkle, John Morris, David Holtzman, Richard Perrin, Adam Naj, Jonathan Haines, Gerard Schellenberg, Victoria Fernandez, Christiane Reitz, Gary Beecham, Alzheimer’s Disease Genetics Consortium, Charles F. and Joanne Knight Alzheimer’s disease research center Knight ADRC

**Affiliations:** Washington University, School of Medicine; Washington University in St. Louis; Department of Psychiatry, Washington University School of Medicine, St. Louis, MO, USA; Washington University School of Medicine; University of Miami; Washington University in St. Louis; Columbia University; Columbia University; University of Miami; Washington University School of Medicine; Washington University School of Medicine, St. Louis; University of Miami; University of Miami; The John P. Hussman Institute for Human Genomics, University of Miami, Miami, Florida; University of Miami School of Medicine; Knight Alzheimer Disease Research Center; Washington University School of Medicine; Washington University in St. Louis; University of Pennsylvania; Case Western Reserve University; University of Pennsylvania; Washington University in St. Louis; Columbia University; University of Miami; University of Pennsylvania School of Medicine; Charles F. and Joanne Knight Alzheimer’s disease research center

## Abstract

Alzheimer Disease (AD) is a highly polygenic disease that presents with relatively earlier onset (≤70yo; EOAD) in about 5% of cases. Around 90% of these EOAD cases remain unexplained by pathogenic mutations. Using data from EOAD cases and controls, we performed a genome-wide association study (GWAS) and trans-ancestry meta-analysis on non-Hispanic Whites (NHW, NCase=6,282, NControl=13,386), African Americans (AA NCase=782, NControl=3,663) and East Asians (NCase=375, NControl=838 CO). We identified eight novel significant loci: six in the ancestry-specific analyses and two in the trans-ancestry analysis. By integrating gene-based analysis, eQTL, pQTL and functional annotations, we nominate four novel genes that are involved in microglia activation, glutamate production, and signaling pathways. These results indicate that EOAD, although sharing many genes with LOAD, harbors unique genes and pathways that could be used to create better prediction models or target identification for this type of AD

## 1. Introduction

Alzheimer Disease (AD) is a highly polygenic disease that often affects individuals over the age of 65–70 years but can affect individuals as young as 30 years old (yo)^1^. Many of the known genomic loci influencing AD have been discovered through large-scale genome-wide association studies (GWAS) and family studies^2^ mainly studying late-onset AD (LOAD). While LOAD dementia^3^ is more common, the earlier onset form of AD (EOAD) makes up 5%−10% of all AD cases^4^. Additionally, it has a higher heritability^5^, has a more aggressive progression and presentation^6,7^, and often has a more severe impact on life and family. This is especially the case for high-risk populations such as racial and ethnic minorities^8^. Despite this, EOAD remains largely understudied^9^.

EOAD is characterized by AD onset before the age of 65–70 yo^5,10^, but this age cutoff is arbitrary and does not appropriately reflect the underlying biology^9^. In practice, age thresholds vary (often 60, 65, or 70)^11^ depending on the scientific question being studied. Previous studies have identified autosomal dominant mutations in well-established AD genes, *APP, PSEN1*, and *PSEN2*^7,12–16^, which cause AD, often presenting with earlier-onset; however, such mutations are rare and are only present in about 10% of EOAD cases^17^. Given the limited genomic studies on EOAD, the genetic etiology of EOAD remains unclear. It is not clear if EOAD is genetically distinct from LOAD; if they are genetically identical, but with a spectrum of onset; or if there some but incomplete overlap.

The AD knowledge gap is further burdened by a lack of ethnic and racial diversity in studies^9^. One of the largest AD-risk GWAS of nearly 800,000 non-Hispanic white (NHW) participants, published by Bellenguez et al.,^18^ (referred to from here on as “Bellenguez”) uncovered 75 genomic loci influencing AD risk, 42 of which were novel. However recent studies that include non-European ancestries but with significantly smaller sample sizes identified novel loci additional loci. One such example is a recent LOAD GWAS of nearly 8,000 (2,748 cases, 5,222 controls) African Americans (AA), performed by Kunkle et al.,^19^ (referred to from here on as “Kunkle”). Kunkle identified one novel genome-wide significant locus and ten novel disease associated loci compared to Bellenguez^18^. A more recent study of 9,168 AA was performed by Ray et al.,^20^ (2,903 cases, 6,265 controls) as a follow-up of the analyses by Kunkle. Ray et al., identified 12 additional novel disease associated loci, including one at genome-wide significance. In the most recent large-scale GWAS for 8,036 (3,962 cases, 4,074 controls) Japanese participants^21^ (referred to from here on as “Shigemizu”) a novel ancestry-specific susceptibility locus near *FAM47E* was identified. Additionally, in a recent trans-ancestry meta-analysis with the 2013 IGAP study^22^, Shigemizu discovered a novel trans-ancestry susceptibility locus near *OR2B2*^21^. Lastly, Sarnowski et al., recently carried out an ancestry-specific GWAS of circulating total tau levels in datasets of 14,721 NHW and 953 AA participants^23^. They identified three tau-associated loci in the AA dataset that were not replicated in NHW datasets. Furthermore, across these four non-NHW studies, besides *APOE*, only ten loci overlap with the now 80 discovered in NHW datasets. In addition, in a recent multi-ethic meta-analysis of 56,241 ADGC participants by Rajabli et al.,^24^ trans-ancestral meta-analyses identified two novel loci, and Lake et al.,^25^ (n = 644,188) identified two novel disease associated loci by meta-analysis of summary statistics from five recent LOAD GWASs of various ancestries and a de novo GWAS of Caribbean Hispanics. Taken together, these studies clearly demonstrate that there is missing biological information surrounding AD that cannot be addressed solely by increasing sample size. Studies of multiple ancestral ancestriers are, thus, critical to identify AD-associated variants not present in NHW.

We hypothesize that by performing multi-ancestry EOAD GWAS, we will identify novel loci that will not be uncovered by studying only Europeans. In addition, as we and others have demonstrated that EOAD is enriched in genetic risk factors, which should lead to more statistical power. This will lead to identify novel loci as well as to determine the overlap and unique genetic architecture of EOAD compared to LOAD. To this end, we utilized genetic data of over 70,000 participants in three ancestries —NHW, AA, and East Asian (Asian)— from the Alzheimer Disease Genetics Consortium (ADGC) and the Charles F. and Joanne Knight Alzheimer Disease Research Center (Knight-ADRC) at Washington University in St. Louis to perform single variant analysis, trans-ancestry meta-analysis, and fine-mapping for gene identification in EOAD.

## 2. Results

### 2.1 Analysis Datasets

**Figure 1:**
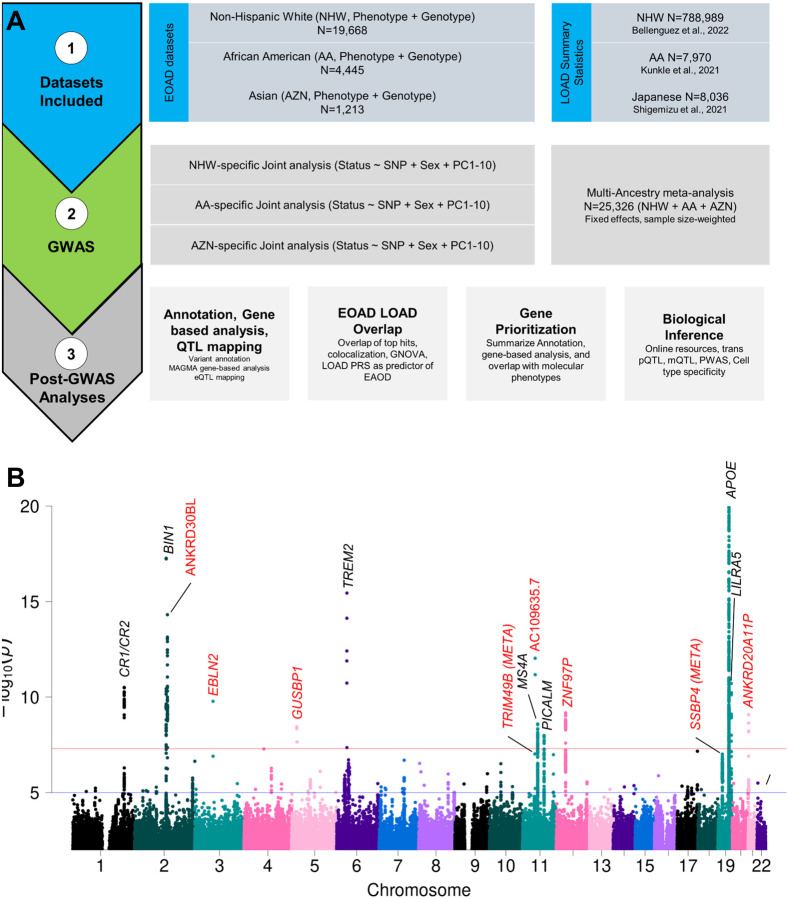
Project workflow and Meta-analysis This project analyzes three ancestries; non-Hispanic Whites (NHW), African Americans (AA), and Asians to identify genes and genomic loci associated with earlier onset Alzheimer disease following the workflow in panel A. Shown in panel B is the Manhattan plot of the trans-ancestry meta-analysis. Loci are annotated with the nearest gene to the top hit. The blue-horizontal line represents the suggestive p-value threshold of 1×10^−05^, and the red-horizontal line is the genome-wide significance threshold of 5×10^−08^. Novel loci are highlighted by red text.

This project uses genetic and phenotypic data from participants from the ADGC and Knight-ADRC who are self-described as either NHW, AA, Asian, or Hispanic (HIS). Imputation was performed using the TOPMed imputation server (GRCh38; Materials and Methods 4.2). Principal component analysis (PCA) was used to group subjects based on genetic similarity to populations of known ancestry (Figure S1A). Datasets were restricted to participants with of age at onset (AAO) ≤ 70 yo for EOAD and age at last visit > 70 yo for controls, to maximize statistical power across datasets. Sensitivity analyses, restricted to AD cases with age at onset ≤ 65, were also performed. The final analysis datasets consisted of 19,668 NHW (6,282 CA, 13,386 CO), 4,445 AA (782 CA, 3,663 CO), and 1,213 Asian (375 CA, 838 CO). No analyses were performed for the ADGC Hispanic dataset due to the limited sample size at time of analysis (n = 550). A summary of the analysis workflow is displayed in Fig. 1A. Subject demographics are summarized in Table S1 for all subjects of each ancestry and is broken down by cohort in Table S2.

### 2.2 Single variant and multi-ethic meta-analysis

Within-ancestry single variant analysis identified 13 genome-wide significant loci across AA, Asian, and NHW. All 13 loci were identified in the NHW analysis (Figure S2A), while for AA (Figure S2B) and Asian (Figure S2C) only the locus near *APOE* —driven by the *ε4* variant— was identified. Seven of the 13 novel loci are located near *CR1, BIN1 TREM2, MS4A, PICALM, APOE*, and *LILRA5*— and were previously identified in LOAD^18,19,21,22,26^; the remaining six loci were novel (Figure S2 and Table 1). AS this study includes dataset from multiple cohorts and arrays, we performed sensitivity analyses by cohort to confirm the analyses and determine potential heterogeneity for each locus. Analyses for each of these loci separated by cohort (40 NHW cohorts, 21 AA cohorts) only found significant heterogeneity (logP<−1.30103) for the *APOE locus* (logP_NHW_=−6.662, logP_AA_=1.532). For all the other sings, many cohorts contributed to these associations (Figure S3-S6, Table S4), and there was no significant heterogeneity for any of the loci besides *APOE*, suggesting low underlying variation between studies.

For subsequent trans-ancestry meta-analyses, we used the summary statistics from all three ancestries in a random effects model in Plink v1.9. Meta-analysis identified two additional novel loci near *TRIM49B* (P_Meta_ =1.02×10^−8^) on chromosome 11 and *SSBP4* (P_Meta_ =4.44×10^−9^) on chromosome 19 (Fig. 1B, Figure S7, Table 1). Both AA and NHW contributed to the signal for *SSBP4* (P_AA_=0.021, OR_AA_=1.197; P_NHW_=1.30×10^−7^ , OR_NHW_=1.392; Table 1), but only NHW contributed to the *TRIM49B* signal. To determine how robust the analyses were, we also performed three additional meta-analysis models to determine how robust the results were.. The additional models included I) an inverse-variance weighted, fixed effects model in Metal; II) a basic fixed-effects meta-analysis in Plink; and III) meta-regression with MR-MEGA, which was developed to be robust against heterogeneous effects. There were no additional loci, novel or known, identified from these other models, but both of the identified novel loci near *TRIM49B* and *SSBP4* were genome-wide significant regardless of the model used (Table S3).

There were no genome-wide or suggestive associations with genetic variants in known causal Mendelian dementia genes (*APP, PSEN1, PSEN2*, and *GRN*; Figure S8-S9), suggesting that common variants in these genes do not significantly contribute to sporadic EOAD, or that we do not have enough power.

To identify additional independent signals at each locus, we then performed step-wise conditional analyses. The top hit at each ancestry-specific locus was included as a covariate in single-ancestry analyses restricted to the genomic region at one megabase (1MB) flanks to the GWAS signal. Each subsequent genome-wide significant variant was included in the model at each locus until significance was attenuated (P ≥ 5×10^−8^). Independent signals were identified in four of the NHW GWAS loci (Figure S10-S13, Table S3). In known loci, we identified one additional independent signal in NHW for the *TREM2* locus (Figure S10). The original GWAS locus was driven by the *TREM2* p.R47H variant, while the second independent signal is driven by the p.R62H variant. Additionally, there were eight independent signals in the locus near APOE (Figure S12). These, notably, are also independent of *APOE4* (rs429358) and *APOE2* (rs7412) as none are in linkage disequilibrium with those variants. At the novel loci, we identified one independent signal at the locus near *ANKRD30BL* (Figure S10C-D) and two independent signals at the locus near *AC109635.7* (Figure S11A-C) in NHW. No independent signals were detected in AA or Asian (Figure S11D-E), likely due to lack of power.

Given the differing age thresholds for EOAD seen in AD literature (60, 65, 70)^5,9,10^ we performed a sensitivity analysis for NHW only including cases with an onset 65 yo or younger (CA65). Asian and AA were excluded due to limited sample size and *APOE* being the only GWAS result. The new age trehsoild leads to a decrease of the number of cases in a 49% decrease. We compared the effect sizes of the sentinel passing the suggestive P value threshold, P < 1×10^− 05^ (73 variants). Effect sizes (R^2^ = 0.958, P = 6.74×10^− 51^, Figure S14A) were well correlated between our main analysis and the CA65 analysis and all betas were in the same direction, suggesting the results including the cases with onset below 70 is similar to those from 65.

Finally, we run another sensitivity analyses by removing array as covariate, as this model can lead to overcorrection as in some instances there may be a collinearity between array and cohort, which is also included as co-variate. We found effect sizes were very strongly correlated (R^2^ = 0.960, P = 1.74×10–51; Figure S14B,). However, this model identified two additional novel loci near *MSMB* (chr10:47025293) and *IGLV* (chr22:22690934; Table S3). These results suggest the model without array can identify additional loci which may have been missed by our default model. However, in order to be conservative, and present the more robust results the post-GWAS analysis (below) were done with he model included array as covariate.

### 2.3 Overlap of EOAD and LOAD

Another goal of this project was to understand the degree of overlapping genetic architecture between EOAD and LOAD. As described in the previous section, we identified seven genomic loci which were previously identified in LOAD GWAS by Bellenguez^18^, Kunkle^19^, or Shigemizu^21^ at genome-wide significance (Table S3). Specifically, the lead variants near *CR1, BIN1, TREM2, MS4A*, and *PICALM* in the NHW EOAD analyses were also significant in Bellenguez^18^, that also showed strong colocalization (PP.H4 > 0.984; Table 1, Table S3 and Table S5). The lead variant in the AA and Asian *APOE* locus, the *APOE* ε4 variant, was significant in all LOAD and EOAD analyses. The summary statistics from Bellenguez et al., did not included many variants in the *APOE* locus and therefore no therefore colocalization analyses could not been performed.

Additionally, it should be noted that signal in AA at the *BIN1* locus colocalize with that form Bellenguez^18^, although this locus did not pass the genome-wide threshold, but reached nominal significance in both AA (P = 1.07×10^− 05^, OR = 1.400; Table 1). This in combination with the main GWAS results suggests that *BIN1* might be a risk locus for EOAD in AA as well, but we lack the power to detect it. We also noted that our limited sample sizes for non-white ancestries may limit our power to detect other loci which might otherwise reach significance. However, these results suggest shared loci between EOAD and LOAD are likely driven by the same variants.

For the novel loci, only variants near *TRIM49B* and *SSBP4* loci were nominally significant in Bellenguez (P = 0.022 and 0.043, respectively; Table S3)^18^ and the lead variant near *AC109635.7* was nominally significant in Kunkle (P = 0.029; Table S3)^19^. We believe these findings reinforce the idea that EOAD has at least a partially unique genetic architecture compared to LOAD.

#### 2.3.1 Total genetic covariance between EOAD and LOAD

**Figure 2:**
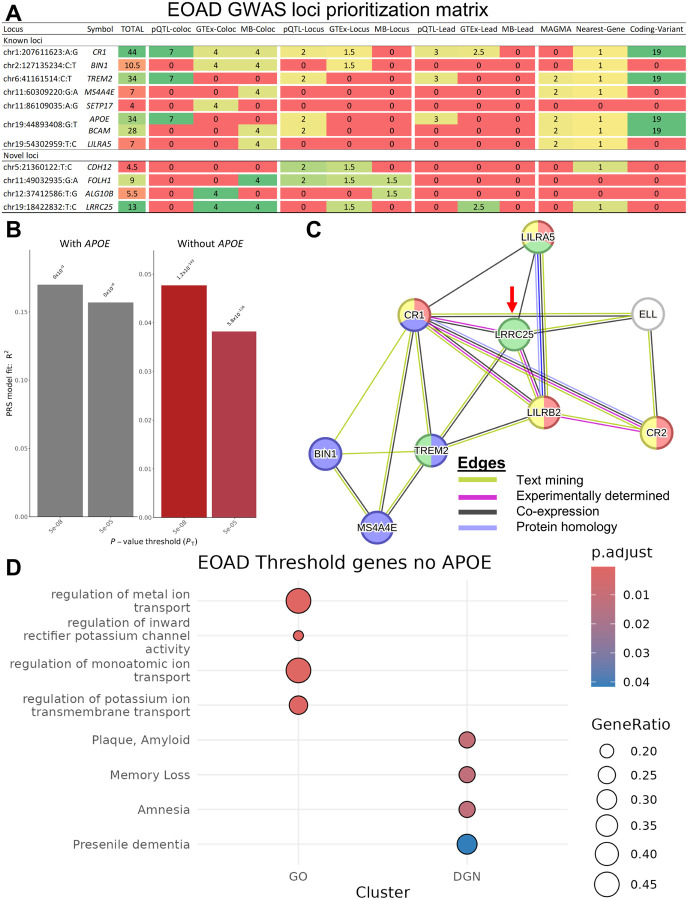
Summary of Prioritized Genes. Matrix of genes prioritized in novel loci (A). Bar plot of LOAD PRS correlation with EOAD status in NHW subjects (B). STRING protein-protein interaction plot of prioritized gene with microglia or immune functions (C). Gene ontology (GO) and disease-gene network (DGN) pathway enrichment results (D) for enriched pathways containing genes prioritized in novel loci.

For a broader view of genetic overlap between EOAD and LOAD, we use two methods to examine the genetic correlation between the two clinical presentations. First, we used polygenic risk scores (PRS) generated with LOAD summary statistics as a predictive variable for EOAD status in logistic regression and determine significant correlations. We found that PRS generated with genome-wide significant SNPs (P ≤ 5×10^− 08^) from Bellenguez^18^ was strongly correlated with NHW EOAD status (R^2^ = 0.170, P < 10^–300^; Fig. 2B Figure S15A). Noting that *APOE* is likely driving most of this association, we also performed the same analysis after removing the *APOE* region (chr19: 44,000,009–47,999,435) from LOAD summary statistics. After removing the *APOE* region, there was still a strong and significant correlation (R^2^ = 0.048, P = 1.20×10^−143^; Fig. 2B, S15B).

Conversely PRS using genome-wide SNPs from Shigemizu^21^ had a very weak correlation with EOAD status in Asians. We did not find strong correlation using Shigemizu PRS: neither with *APOE* (R^2^ = 0.008, P = 0.007) nor without (R^2^ = 0.005, P = 0.036; Figure S15C-D respectively). This may be due to relatively low overlapping variants between Asian EOAD and Shigemizu LOAD analysis^21^ as well as there being only three loci passing the 5×10^− 08^ threshold in Shigemizu’s analysis^21^. Using a relaxed threshold of 5×10^− 05^ to calculate PRS, we see much stronger correlation as the R^2^ increases to 0.085 with *APOE* and 0.084 without *APOE* (P = 1.13×10^−16^ and 2.64×10^−16^ respectively, Figure S15C-D). This suggests that there is, in fact an overlap between LOAD and EOAD in Asian populations but the current sample size is not large enough to determine the extent of the overlap. It should be noted; however, that Shigemizu’s analysis focuses on Japanese datasets^21^, while our Asian dataset largely consists of Japanese participants, there are still other East Asian samples included which may impact the risk score correlation. Finally, PRS using GWS from Kunkle^19^ correlated significantly with EOAD AA (R^2^ = 0.063, P = 1.46×10^−37^) with *APOE* included. However, *APOE* was the only GWS signal in Kunkle^19^, so strong correlation here is to be expected. Correlation was still robust using Kunkle^19^ SNPs at the P value threshold 5×10^− 05^, both with *APOE* (R^2^ = 0.020, P = 1.61×10^−13^, Figure S15E) and without *APOE* (R^2^ = 0.061, P = 5.63×10^−37^, Figure S15F). Overall, the PRS results suggests that there is covariance between EOAD and LOAD in other loci besides *APOE* in NHW. There is also covariance between LOAD PRS and EOAD status in Asians and AA; however, we suspect the limited covariance at lower p-values is due to low sample size in those datasets. Specifically, the relatively low statistical power restricts the number of loci which contribute to PRS. This as well as that there are potentially overlapping subjects between our AA analysis and those used by Kunkle et al.,^19^ makes interpretation of those results difficult.

Our secondary method for investigating total genetic overlap included Linkage Disequilibrium SCore (LDSC) regression. LDSC found strong overall genetic covariance between EOAD and LOAD (rg = 0.791, P = 6.80×10^−12^). From local genetic covariance between summary statistics from Bellenguez^18^ and NHW, we identified regions of significant (P ≤ 0.05) covariance near six previously identified GWAS loci: *CR1* (P = 0.022, corr = 1.196), *TREM2* (P = 3.50×10^− 07^, corr = 1.318), *MS4A* (P = 4.90×10^− 06^, corr = 0.984), *PICALM* (P = 0.027, corr = 0.907), *APOE* (P = 0.036, 0.023, corr = 1.076, 0.983), and *LILRA5* (P = 0.027, corr = 0.702; Table S6). We did not observe any significant local genetic covariance between EOAD and LOAD at the novel loci suggesting the LDSC results are mainly driven by the previously identified loci.

### 2.4. Gene prioritization in GWAS loci

To help determine the causal gene at each locus we prioritized functional genes by creating a 50-point weighted presence/absence matrix (Table S6) summarizing evidence from colocalization with molecular quantitative trait loci (QTL), including CSF protein QTLs (pQTL) and brain expression QTLs (eQTL); locus and lead variant overlap with those same molecular phenotypes; Multi-marker Analysis of GenoMic Annotation (MAGMA)^27^ gene-based analysis, and variant annotation, including the nearest gene and genes with protein-altering variants in our GWS loci. We generated scores for all genomic features (genes, long non-coding RNA, and pseudogenes) within 1MB flanks of the top hit in each locus according to the guidelines in Table S6. Genes had to pass a minimum score threshold of four (≥ 4) to be nominated. At each locus, genes that had a ≥ 20%. relative difference compared to the top scoring in the locus were not prioritized.

To internally validate our prioritization methods, we applied our scoring strategy to previously identified AD loci to see if we would prioritize the same functional gene as is suggested by literature. That is, we tested to see if our methods would prioritize *CR1, BIN1, TREM, MS4A4A/MS4A6A*, *PICALM* and *APOE* as literature has suggested these genes to be the functional gene at their respective loci. With regards to the LILRA5 locus, Bellenguez et al.,^18^ prioritized *LILRB2* as a tier 2 gene for the locus based on being the nearest gene and enrichment for expression in microglia. Given the lack of any other GWAS or functional analyses strongly supporting this idea, we did not use this locus as a basis of whether our prioritization methods were successful.

We were able to prioritize causal genes for the previously identified *CR1, BIN1, TREM2*, and *APOE* loci (Fig. 2A, Table S8).

For the novel loci we were able to prioritize four genes for four out of eight loci (Fig. 2A). Specifically, we prioritized *CDH12* (chr5p14.3), *FOLH1* (chr11p11.12), *ALG10B* (chr12q12), and *LRRC25* (chr19p13.11). Notably, *FOLH1* and *LRRC25* are prioritized within our two novel trans-ancestry loci. *CDH12* is a cell adhesion gene expressed in vesicles and plasma membrane that functions in cell adhesion^28,29^. *CDH12* was the sole gene prioritized in the novel chr5:21360122 locus based on evidence from QTL mapping and annotation. Specifically, the GWAS locus overlapped significant cis-pQTL (Fig. 2A, Table S9) and cis-eQTL (Fig. 2A, Table S10) loci, as well as it is mapped as the nearest gene to at least one genome-wide variant in the locus by FUMA. *FOLH1* is expressed extracellularly and in plasma membrane. It has specific brain functions for the modulation of excitatory neurotransmission, particularly as it relates glutamate activity^28,29^. It was the second highest scoring gene of the four, having MetaBrain eQTLs that colocalized (Fig. 2A, Table S11) with the GWAS locus (chr11:49032935). While the colocalization evidence alone was enough to prioritize this gene for this locus, it is additionally strengthened by having genome-wide significant loci for all three assessed molecular QTLs overlapping the GWAS locus (Table S12). *ALG10B* is primarily localized to the endoplasmic reticulum (ER) and functions in transferase activity and protein metabolism^28,29^. Like *FOLH1*, this gene was supported by GTEx eQTLs that colocalize with the GWAS locus, which alone was sufficient for its prioritization. The only other evidence for this gene was an overlapping locus with MetaBrain eQTLs. Lastly, there is *LRRC25* which functions in NF-kappa-B signaling pathway and type I interferon signaling pathway inhibitions^28,29^. Its expression is localized to the cytosol, ER, and microtubules. This gene had the most supporting evidence for its prioritization. Functional evidence for this gene includes GTEx and MetaBrain eQTLs colocalizing with the GWAS locus, a genome-wide significant eQTLs within the locus, and the lead SNP (chr19:18422832) is itself a genome-wide significant eQTL in GTEx. The gene, *ELL*, was also a candidate for prioritization as GTEx eQTLs for the gene colocalized with the GWAS locus (Table S8, Table S10). It does not have as much supporting evidence, but the colocalization result suggests it could be a secondary actor in the locus for influencing EOAD.

### 2.5 Biological insights for GWAS loci and prioritized genes

To further disentangle the biological relevance of these of prioritized genes in our GWAS loci, we leveraged data from five broad categories: I) brain cell-type specificity, II) expression in AD vs Controls, III) protein-based evidence, IV) associations with other phenotypes, and V) enrichment in biological pathways. The findings of these analyses are summarized in Fig. 2C–D.

#### 2.5.1. Evidence for microglia activation and autophagy in novel prioritized genes

We used cell-type specificity data from Western et al.,^30^ to query our prioritized genes for a primary cell-type in brain tissues (Methods 4.7.1). We particularly took note of any genes which were enriched in microglial expression because several genes known to affect AD (e.g., *CR1, MS4A, HLA*, and *TREM2*)^2,31–34^ are microglia genes involved in pathways related to amyloid beta (Aβ) clearance or autophagy, as is *LRRC25*. In addition to its functional role described above, *LRRC25* regulates virally induced autophagy. It was also highlighted as an AD risk gene by Kosoy et al.,^35^, so it may serve a similar phagocytotic function as known microglia-specific AD risk genes. To investigate this hypothesis, we leveraged STRING^36^ protein-protein interaction (PPI) analysis and brain tissue expression data from Agora to identify a functional relationship between LRRC25 and known AD microglial genes. In our PPI analysis, we queried genes which passed the score threshold for prioritization (Table S8) in known and novel GWAS loci and used all protein coding genes as the background set. However, due to pleiotropy within the *APOE* locus, genes from that locus were not included. STRING identified significantly more interactions than expected by chance (PPI enrichment P = 2.36×10^− 10^). Notably there were text-mining, experimentally determined, and co-expression edges shared between *LRRC25* and *CR1/CR2, TREM2, LILRA5* and *LILRB2*, all of which have microglia related functions in AD (Fig. 2C, Figure S16A). It should be noted that we see similar edges between *ELL* and *CR1/CR2*, we do not, at this point, suspect it functionally affects AD through microglia-mediated functions. In support of the PPI findings, we also found from Agora expression data that the Log2-fold change (L2FC) for *LRRC25* (0.47) was nearly identical to that of *CR1* (0.36) in AD cases compared to controls in two brain tissues— the inferior frontal gyrus (IFG) and parahippocampal gyrus (PHG), which is not observed novel-known gene pairing in the same tissue (Table S13). This result supports the PPI finding of a co-expression relationship between the two genes as well as add tissue localization context for their relationship. Further supporting the role of microglia and autophagy in EOAD are the results from our colocalization between EOAD GWAS loci and trans-pQTLs. This analysis was used to determine which protein might have a distal functional relationship with our GWAS loci as was done for *MS4A* and s*TREM2* by Deming et al.,^32^. The results of our analysis exclude pQTLs which colocalize with the APOE locus, as it is known to have broad pleiotropic effects.

We found EOAD variants in the *BIN1* locus colocalized with a trans-pQTLs for *TMEM132C* (Table S14). *TMEM132C* codes for trans-membrane protein 132C whose expression is enriched in oligodendrocytes^37^ and localized to cytosol and centrosome^38^ according to data sourced from proteinatlas.org. There are no functional annotations which elucidate how the *BIN1* locus might be impacting AD through regulation or modification of *TMEM132C*, however, *TMEM106B* is a known dementia-associated gene which has functions in lysosome function and homeostasis. Being from the same family of genes*, TMEM132C* may work alongside *BIN1* in autophagy pathways^39^. Specifically, one study has found that *BIN1* contributes to early endosome size deregulation^40^, and that may be due attributed to pQTLs near *BIN1* modifying *TMEM132C* function. In addition to microglia and autophagy pathways, animal studies have found that *BIN1* modulates glutamate activity^41^, which we believe is an alternative pathway contributing to EOAD.

#### 2.5.2 Novel prioritized gene involvement in glutamate dysregulation

In addition to microglia-mediated autophagy pathways, neurotransmitter dysregulation can also impact AD onset and progression. Specifically, glutamate is an excitatory neurotransmitter which can be neurotoxic at high levels and is found to be dysregulated in AD^8,42–44^. Information from public resources suggest that *FOLH1* contributes to the modulation of glutamate so we leverage proteomic data, mRNA brain expression information from Agora, pathway analyses, and associations with other phenotypes to find evidence supporting the hypothesis that *FOLH1* affects EOAD through its modulation of glutamate. In proteomic analyses we use pQTL data from Western et al.,^30^ for colocalization analsyes, proteome-wide association study (PWAS) and Mendelian randomization on our prioritized genes. While the former two analyses provided no significant results for any novel prioritized genes (Figure S16B), we do find that *FOLH1* was significant in MR analysis when using pQTL as instrument variables (P = 8.00×10^− 07^; Table E2) and thus have a likely causal effect on EOAD. Based on data from Agora, it is significantly upregulated in parahippocampal gyrus (PHG) of cases compared to control. Because of the positive relationship between *FOLH1* and glutamate – that is, it hydrolyzes N-aceylaspartylglutamate to release glutamate^45^ – this result suggests that *FOLH1* is causing more glutamate to be excreted in that brain region and causing a neurotoxic effect. Notably, *FOLH1* is significantly downregulated in cases compared to controls in anterior cingulate cortex (ACC) and cerebellum (CBE). We can speculate that this difference indicates differences in Amyloid beta (Aβ) presence in those tissues. We suspect that is the case because *TREM2*, which recruits microglia for Aβ clearance^46^, is also downregulated in CBE in cases compared to controls, while it is upregulated in PHG. Studies have suggested that Aβ contributes to synaptic failure through modifying the glutamate related systems^44^. Thus, lower levels of *FOLH1* and *TREM2* may indicate a lack of Aβ accumulation in those tissues. These speculations are consistent with our pathway analysis results which found that *FOLH1* is enriched in disease-gene network analysis for memory impairment, amyloid plaque, memory loss, presenile dementia, and memory impairment (Fig. 2D, Table S15).

Our biological analyses also implicate ion flow dysregulation as a potential pathway driving EOAD. Specifically, the data suggests calcium ion (Ca^2+^)^47^ and potassium ion (K^+^)^48^ dysregulation may contribute to glutamate dysregulation and subsequent neurotoxicity. This is supported by several points evidence. First, we see calcium transport, calmodulin binding, and calmodium-dependent signaling pathway enrichments for known AD genes *BIN1, TREM2, LILRA5*, and *LILRB2* (table S15). Calmodulin acts downstream of glutamate production by binding Ca^2+^ ions which pass through the glutamate activated NMDA receptor^47,49^. NMDA receptors are expressed on glial cells and can induce pro-inflammatory responses^50^. This increased activation NMDA receptors may additionally lead to downstream signaling changes such as inactivating extracellular signal-regulated kinases (ERK)^49^, which may explain our *CDH12* association. *CDH12* codes for the Cadherin 12 protein which functions in calcium-dependent cell adhesion and is implicated in synaptogenesis, cell junction organization, and ERK signaling^28,29^. According to data from Agora, *CDH12* is significantly downregulated in seven AD-relevant brain regions (Table S13), so this is consistent with expectations given lower ERK signaling^51^. Regarding K^+^ dysregulation, we also see enrichment in potassium transport pathways implicating *TREM2, BIN1*, and our novel prioritized gene *ALG10B* (Table S15). Glutamate transporter genes, such as *GLT1*, function by co-transporting three sodium ions and one hydrogen ion into the cell, while counter-transporting one potassium ion^52^. Dysfunction of *ALG10B*, which normally functions in glycosylation^53^ and inward rectifier potassium channel regulation (Fig. 2D, Table S15, Figure S16), may lead to an inability of glial cells to transport glutamate out of the post synaptic space, leading to neurotoxic accumulations.

#### 2.5.3 Protein-proteins interactions for novel prioritized genes in EOAD

To further elucidate potential latent pathways associated with EOAD, we also tested for complex gene interaction and if any modifiable risk factors are associated with our prioritized genes and with AD. To support this direction, we look at results from trans-pQTL colocalization, GWAS catalog associations with AD risk factors, and metabolite QTL (metabQTL^54^) colocalization.

Next, we queried the GWAS catalog (https://www.ebi.ac.uk/gwas/)^55^ for other phenotypes that implicate prioritized genes in our novel loci. Specifically, we selected all genes which were within our novel GWAS locus and which had a prioritization score greater than or equal to four (≥ 4). We then searched for those genes within “mapped gene” and “reported gene” fields from the GWAS catalog results^55^. We additionally noted if any of those phenotypes were in one of four AD relevant categories: I) dementia; II) AD biomarkers such as *TREM2* or *Tau*; III) other neurological or psychiatric traits such as depression, stroke, or schizophrenia; and IV) AD risk factors such as, cancer, heart disease, or diet. Starting with dementia associations, we found that variants mapped to *LRRC25* were previously reported to be associated with AD-related phenotypes (Table S16), specifically with GWAS of upper vs lower quantiles of AD PRS (rs754032589)^56^ and a GWAS of AD and gastroesophageal reflux disease (rs3859570; Table S16)^57^. Looking at AD biomarker associations, variants in the *CDH12* gene region have been reported to be associated with PHF-tau measurement (rs2516478/rs1261246 and rs10805748/rs2250562)^58^. Next, we found that rs61350355 in the *FOLH1* locus is associated with amygdala volume change rate^59^, rs147303113 (LRRC25) is associated with brain shape measurement^60^, and variants mapped to *CDH12* are associated with cognitive domain measurement (chr5:22528391)^61^, cognitive function measurement (rs183856)^62^, and schizophrenia (rs11738207 and rs2680786^63^; Table S16). Finally, variants mapped to each of the novel loci prioritized genes are associated with 21 phenotypes across 10 unique modifiable AD risk factors including alcohol consumption, blood pressure, cancer, cardiovascular disease, diabetes, diet, educational attainment, infection, lipid measurements, and body weight (Table S16). GWAS catalog hits mapped to *LRRC25* and *FOLH1*^55^ are correlated with our GWAS signal, adding confidence that these functions are linked for those genes. We did not find similar correlations for *CDH12* or *ALG10B* mapped variants, so more work needs to be done to validate the function of these loci to EOAD.

Lastly, colocalization with CSF metabolites QTLs^54^ identified 13 unique metabolites which had metabQTLs colocalize with three EOAD loci. Eleven of the colocalization results were driven by the *APOE* locus (Table S17). The remaining two results were colocalization between myo-inositol metabQTLs and the SSBP4 locus, and colocalization between metabQTLs for an unnamed metabolite internally referred to as “X-13684 with the chr5:21360122 locus using NHW summary statistics (Table S17). The colocalization result with myo-inositol is likely driven by *ELL*, which is linked to myo-inositol levels in the GWAS catalog^55^ (Table S16). Myo-inositol is synthesized in high concentrations in the brain where it plays a role in facilitating the binding of neurotransmitter and some steroid hormones to their receptors. This may suggest that *ELL* plays a similar role to *FOLH1* in EOAD. Furthermore, a query of The Human Metabolon Database found that myo-inositol levels are reduced in patients with depression^64^, which can be a symptom of AD. One other study has previously investigated a link between myo-inositol levels and AD but did not find a significant difference in the metabolite levels between cases and controls^65^. Our results may indicate that the relationship between myo-inositol and AD is earlier-onset specific.

## 3. Discussion

The goal of this study was to identify novel genes and genomic loci associated with EOAD and to determine the degree of overlap between the genetic architectures of EOAD and LOAD. Using genetic and phenotypic data from 19,668 NHW, 4,445 AA, and 1,213 Asian participants from ADGC and Knight-ADRC, we performed the largest multi-ancestry GWAS of EOAD to date. Our analysis identified eight novel loci, including two of which were only identified in trans-ancestry meta-analysis (Fig. 1B).

A total of seven loci were already previously identified in at least one of the LOAD analyses by Bellenguez^18^, Kunkle^19^ or Shigemizu^21^. On the other hand, five of the eight novel EOAD loci were not observed even at nominal significance in previous LOAD analyses (Table S3). Those which were at nominal significance include the trans-ancestry loci near TRIM49B and SSBP4, which were nominally significant in Bellenguez^18^ (P = 0.022 and P = 0.043, respectively), and the NHW locus near *AC10635.7*, which was nominally significant in Kunkle (P = 0.029)^19^. This suggests that EOADs underlying genetic architecture is at least partially unique from LOAD and genes in these novel loci either are not contributing to LOAD risk or current studies have not been able to capture them. The lack of overlap between LOAD and EOAD is possibly a consequence of LOAD having more age-related concomitant health issues and overlap with other late-onset dementias than EOAD.

Through colocalization results, we found overlap at previously identified loci near CR1, BIN1, TREM2, PICALM and APOE, suggesting they are likely to share causal variants in EOAD and LOAD. The colocalization at these loci is likely to be driving the overall genetic overlap between LOAD and EAOD (LDSC rg = 0.791). At the same time, this finding further suggests that our novel loci indicate that EOAD has a partially distinctive underlying genetic architecture from LOAD. Additionally, we also saw strong correlation between EOAD status and LOAD PRS in NHW, even without *APOE* (Fig. 2B), which was expected given the number of overlapping loci and the LDSC results. However, LOAD PRS results for AA and Asian suggests that Japanese and AA LOAD risk studies do not have sufficient power to detect many loci that can be used for PRS. Taking from the results in NHW, we propose that genes in previously identified loci are likely strong drivers of all AD, but novel loci are likely contributing to differences seen between the two AD types. Future EOAD-specific analyses and larger trans-ancestry analyses will be important to confirm this finding.

To put our trans-ancestry results in context with more recent findings and assess our hypothesis, we can compare our findings and conclusions with trans-ancestry LOAD analyses by Lake et al.,^25^ and Rajabli et al.,^24^. Lake et al., were able to identify two novel trans-ancestry LOAD loci by meta-analysis of summary statistics from five recent LOAD GWASs; from Bellenguez^18^, FinnGen Release 6 (https://www.finngen.fi/en/access_results), Shigemizu^21^, Kunkle^19^, and a *de novo* GWAS of Caribbean Hispanics (Ncases = 54,233, Nproxy-ADD = 46,828, Ncontrols = 543,127). We checked to see if any peaks from Lake et al., or Rajabli et al., overlapped with this EOAD analyses, but none of the top hits from those analyses passed nominal significance in EOAD analyses. This reinforces the premise of EOAD and LOAD having at least partially distinct genetics and adds that this is consistent within and across ancestry. However, while there were no GWAS results in common with our analysis, we do find some gene and pathway overlap. Specifically, Rajabli et al., propose the nearest gene to one of their GWAS loci, *LRRC4C* as a potential AD functional gene. *LRRC4C* is part of the same leucine-rich repeat family of genes as our prioritized gene *LRRC25. LRRC4C* has been found to be implicated in neurodevelopmental disorders including developmental epileptic encephalopathy^28,29^. This may implicate this family of genes as having important latent functions which impact AD. Additionally, previous studies implicate insulin receptor activity regulation by *GBR14*^24^. This is in line with our findings of a relationship between *ELL* and myo-inositol in EOAD (Table S17), as myo-inositol also modulates insulin-mediated signaling. We believe it would be difficult, if not impossible, to identify these genes and pathways by large-scale analysis of NHW populations alone or without comparisons of EOAD and LOAD. This reinforces the necessity for more and larger GWASs of EOAD vs LOAD and broad ancestral origins.

Lastly, we nominated what we believe are the likely functional genes in four novel GWAS loci; CDH12 at the chr5:21360122 locus, *FOLH1* at the chr11:49032935 locus, *ALG10B* at the chr12:37412586 locus, and *LRRC25* at the chr19:18422832 locus. The combination of our genetic and biological results suggests that *FOLH1* and *LRRC25* are our strongest candidate genes for affecting EOAD (Fig. 2A), but we *CDH12* and *ALG10B* are important downstream elements which exacerbate neurodegeneration. Both *LRRC25* and *FOLH1* had strong genetic evidence from colocalization and shared loci GTEx and MetaBrain eQTLs. Biologically, *LRRC25* is enriched for expression in microglia, a cell type which is known to be functionally relevant in AD^18,35,66^. It has functions in NF-kappa-B and type I interferon signaling pathway inhibition and virally induced autophagy, so it is likely contributing to EOAD through known microglial activation and autophagy pathways. This is supported by results from STRING, which suggests that it is enriched for interactions with *CR1*, *TREM2, LILRA*, and *LILRB2*, all of which function in similar pathways. Additionally, *LRRC25* follows a similar expression pattern to *CR1* in parahippocampal gyrus (PHG) and superior temporal gyrus (STG; Table S13), so it is likely affecting EOAD through its relationship to *CR1* in those brain regions. Lastly, it may also be affecting EOAD through a functional relationship with *ELL*, which is enriched in LOAD and tauopathy in STRING human phenotypes. On the other hand, its interaction with myo-inositol, as suggested by metabQTL colocalization (Table S17) and GWAS catalog results (Table S16), indicates it may affect EOAD through its relationship with neurotransmitters as some can be neurotoxic in high concentrations. That neurotransmitter interaction may also mean *ELL* is related to *FOLH1*. As described in the results, *FOLH1* is enriched for expression in oligodendrocytes and functions in modulating excitatory neurotransmission, specifically glutamate activity^45^ which is neurotoxic in levels. *FOLH1* is significantly higher expressed in PHG of AD cases compared to controls so this may be what drives the relationship between this gene and AD. Furthermore, it is enriched in disease-gene networks for memory loss and amyloid plaque, as well as it is associated with multiple glutamate phenotypes and amygdala volume change rate in the GWAS catalog^55^, giving us high confidence that this gene is associated with AD via this pathway (Table S15). Finally, our analyses suggest that glutamate dysregulation may also be driving CA^2+^ and K^+^ ion flow dysregulation. Glutamate release into the synaptic space drives CA^2+^ transport into NMDA receptors^47,49,50,67,68^, which in turns modulates calmodulin-binding and signaling pathways which are downstream of it^47,49^. These downstream pathways include ERK signaling, which is modulated in part by *CDH12*^51^ and NF-kappa-B signaling, which is modulated in part by *LRRC25*^69^. Glia cells may also fail to transport glutamate out of the synaptic space because of *ALG10B* dysfunction and subsequent dysregulation of potassium channels that enable glutamate uptake^48^.

This study does have some weaknesses that need to be acknowledged. Firstly, despite a similar sample size and single variant analysis model to the work done by Rajabli et al.,^24^, this study identified more novel associations with AD. Given the limited availability of large-scale EOAD analyses for comparison, it is challenging to entirely dismiss the possibility of false positives arising a consequence of our study design. Specifically, one might suspect that our GWAS results are a consequence of genetic variability from the number of cohorts included in the single variant analysis for each ancestry. However, when array, which functions as a partial proxy for cohort, was included as a covariate in our model, there was very little genomic inflation in our single variant analysis (λ_NHW_ = 1.045, λ_AA_ = 1.014, λ_Asian_ = 1.001), and we found no evidence of heterogeneity within novel loci as well as many cohorts contributed to each signal (Figure S4-S6). This, in addition to the fact that we were able to identify prioritized genes for 50% of our GWAS loci, suggests that including ten genetic PCs in the model as well including array as a covariate was sufficient to control variability and gives us confidence that most, if not all, of our signals are likely real. We also must acknowledge that although we aimed to add to the field’s understanding of AD biology by including multiple ancestries, we lacked the power to identify novel loci specific to AA or Asian. However, including those ancestries allowed us to identify two novel trans-ancestry signals and we had sufficient evidence from molecular traits to prioritize strong candidate genes for those loci.

In summary, we performed the largest trans-ancestry GWAS of EOAD to date, identifying eight novel ancestry-specific and trans-ancestry loci for this form of AD. We established lines of similarity and distinction between EOAD and LOAD based on ours and other large-scale AD GWAS analyses. We were able to use proteomic, transcriptomic, and metabolomic data to identify likely functional genes in our GWAS loci and we highlight *FOLH1* and *LRRC25* as likely driving EOAD through microglia activation/autophagy pathways, and dysregulated excitatory neurotransmitter function, respectively.

## 4. Materials and Methods

### 4.1 Cohorts

This project used genotype and phenotype data of participants (n=70,620) who self-identified as either Non-Hispanic White (NHW, n=50,180), African American (AA, n=8,563), Asian (n=4,742), or Hispanic (HIS, n=2,292) from the Alzheimer’s Disease Genetics Consortium (ADGC) as well as participants from the Knight Alzheimer’s Disease Research Center (Knight-ADRC)(n=4,843). The ADGC collects data from multiple genotyping rounds from several studies.

These include the Adult Changes in Thought (ACT) study, the National Institute on Aging (NIA) Alzheimer’s disease centers (ADC), the Alzheimer’s Disease Neuroimaging Initiative (ADNI), The Predictor of Cognitive Decline Among Normal Individuals study (BIOCARD), the Chicago Health and Aging Project (CHAP), the Children’s Hospital of Philadelphia (CHOP), the Einstein Aging Study (EAS), Glaxo Smith Kline (GSK), the Indianapolis Ibadan Dementia Study (Indianapolis), Johns Hopkins University (JHU), JPN2012, Mayo Clinic Jacksonville (MAYO) and Rochester (RMAYO), The Multi institutional Research of Alzheimer Genetic Epidemiology (MIRAGE) study, the Netherlands Brain Bank (NBB), the National Institute of Aging initiative for Late-Onset Alzheimer’s disease and the National Centralized repository for Alzheimer’s Disease and Related dementias (NIALOAD-NCRAD), the Oregon Health & Science University (OHSU), the Rush University Religious Orders Study/ Memory and Aging Project (ROSMAP), the Texas Alzheimer’s Research and Care Consortium (TARCC), the Translational Genomics Research Institute (TGEN), the University of Miami/Vanderbilt University/Mount Sinai School of Medicine (UMVUMSSM), the University of Pittsburgh (UPITT), and the Washington Heights-Hamilton Heights-Inwood Columbia Aging Project (WHIICAP).

Participants from the Knight-ADRC were evaluated by Clinical Core Personnel at Washington University. Cases (CA) were selected on the following bases: a diagnosis of dementia of the Alzheimer’s type, determined by criteria equivalent to the National Institute of Neurological and Communication Disorders and Stroke-Alzheimer’s Disease and Related Disorders Association for probable AD. Severity was evaluated using the Clinical Dementia Rating (CDR). Controls (CO) were assessed by the same criteria and given a nondemented (CDR = 0) diagnosis. Written consent was obtained from all participants.

Phenotypic and covariate data from each study was harmonized and merged. For this study, we selected CA with an age of onset (AAO) on the earlier spectrum of AD: AAO 70 or younger. Conversely, CO were selected as cognitively healthy participants who are older than 70 yo at last assessment. Case-Control status for all ADGC participants were clinically defined following ADRC criteria and clinical dementia rating (CDR, must be ≥ 0.5 for cases) guidelines. To create more homogenous groups among participants, we performed principal component analysis (PCA) for all participants. PCA was performed using Plink v1.9^70^ with only very high quality variants (genotyping rate ≥ 99%, MAF ≥ 0.01, and HWE P>1×10^−06^). Participants were included into analyses for NHW, AA or Asian based on genetic similarity to common genetic ancestries used by HAPMAP. NHW bounds were defined by five standard deviations beyond the means of the first two principal components for trios of Utah residents of northern and western European ancestry (CEU). This was done similarly for AA with respect to Yoruba adult-parent-trios from Ibadan, Nigeria and for Asian with respect to unrelated Japanese individuals from Tokyo, Japan (JPT; Figure S1A). Strict bounds were used to define participants as NHW and Asian since those populations tend to be relatively homogenous. AA and HIS, which are generally more admixed, extend up to the border of NHW and between the borders of NHW and Asian, respectively (Figure S1B). PCA was then performed on each ancestry separately to identify and remove outliers. Finally, to control for cryptic relatedness, we performed Identity by descent (IBD) analysis. Unrelated participants were selected for analysis (Figure S1C) based on pi-hat < 0.198. Within the related pairs, the individual with highest call rate was kept for analysis. After all QC steps, 27,004 (NHW 6,282 CA, 13,386 CO; AA 782 CA, 3,663 CO; Asian 375 CA, 838 CO; HIS 280 CA, 270 CO) participants remained for analysis. Due to low sample size and lack of statistical power, no analyses were performed using the HIS dataset.

### 4.2 Genotype QC

DNA was genotyped on various arrays, mapped to GRCh38 human genome reference, and imputed using the TOPMed imputation server. The following preparation and QC steps were run on the downloaded genetic data; I) the Variant Call Format (vcf) files were converted to plink using PLINK v1.90b6.26. II) the chromosomal plink files of each study were merged for their respective ancestry. III) variants with R^2^ ≤ 0.3^71^ and variants and participants with genotyping rate (GR) < 98% were removed. IV) variants which were not in Hardy-Weinberg equilibrium (HWE, P<10^−06^) were removed from autosomal chromosomes. Autosomal and Sex Chromosome data were then merged back into a single plink file. V) Finally, study-specific plink files for each ancestry were merged into a single, ancestry-specific plink file. Genetic data for the Knight-ADRC participants were generated and processed by the Cruchaga Lab at Washington University in St. Louis (https://cruchagalab.wustl.edu/)with identical QC filters as described in Deming et al.,^32^, including a minimum GR 98% and removal of variants from autosomal chromosomes which are not in HWE (P<10^−06^). After performing QC steps for each ancestry’s phenotype and covariate, we applied a final genotyping rate filter of 90% to maximize the number of high-quality variants as well as minor allele frequency (MAF) filters unique to each dataset based on a minor allele count (MAC) of 5 (NHW MAF ≥ 0.2%;. AA MAF ≥ 0.1%, Asian MAF ≥ 0.02%)

### 4.3. Statistical Analysis

Single variant association analysis was carried out for NHW, AA, and Asian ancestries using plink v2.3. We used a MAC cutoff of five (MAF_NHW_=0.02%, MAF_AA_=0.1%, MAF_Asian_=0.2%), so the number of variants included in each analysis was 12,725,244 for NHW, 19,508,138 for AA and 9,351,864 for Asian. Sex, genotyping array and the first ten principal components (PC) were included as covariates for NHW and AA analyses. Only sex and the first 10 PCs were used for Asian because over 80% of subjects were from a single cohort and genotyped on a single array (Table S2). Age was excluded as a covariate since sample selection is based on age. Additionally, because plink files were merged based on PCA-based ancestry selection, we used a strict HWE filter of P > 1×10^−30^ in the analysis. For each single variant analysis, significance was set at the standard genome-wide significance threshold of 5×10^−08^. Following single variant analysis, meta-analysis was performed with a random-effects model using Plink v1.9 “--meta-analysis” function. Variants from meta-analysis used the same significance threshold as single variant analysis. Following initial single variant analysis, stepwise conditional loci was performed at genome-wide significant loci. In stepwise conditional analysis, the lead variant for each locus was included in the model and analysis was re-run on the locus (1MB flanks on the top hit) to identify independent signals. If an independent signal was identified, the new lead variant was included in the model as well and the steps were repeated until there was no longer any significant signal in the locus.

### 4.4 Annotation and Gene-based analysis

Summary statistics from single variant analysis were annotated using the FUMA^72^ online API after LiftOver^73^ to hg19 format. Multi-marker Analysis of GenoMic Annotation (MAGMA) gene-based analysis^27^, which is integrated into the FUMA web application, was also run using single variant analysis summary statistics for each ancestry as well as meta-analysis summary statistics.

### 4.5 Investigating the shared genetic architecture of EOAD and LOAD

Methods to investigate overlap between EOAD and LOAD include, cross-checking the sentinel hit in GWAS loci, correlation between LOAD PRS and EOAD status, genetic covariance using Linkage Disequilibrium SCore (LDSC)^74,75^ regression local covariance using SUPERGNOVA^76^, and finally, colocalization.

#### 4.5.1 Cross-check of lead SNP in GWAS loci

To cross-check sentinel hits in GWAS loci, we directly compared the P values of the sentinel variant from our EOAD GWAS with the same variant in LOAD summary statistics from the most recent LOAD GWASs of NHW, AA, and Japanese participants.

#### 4.5.2 Correlation of EOAD status and LOAD PRS

Polygenic risk scores (PRS) were calculated using PRSice2^77^ with LOAD summary statistics as the base GWAS and EOAD plink files for the same ancestry as the target. Risk scores were generated for two different models, one including the *APOE* region (chr19; 44,000,009–47,999,435 bp) and one excluding it. PRSice2 was run using LOAD SNPs at the following p-value thresholds; 5×10^−08^, 5×10^−05^, 5×10^−02^, and 5×10^−01^ in each model. The clumping p, R^2^, and kb thresholds were set at 1, 0.1, and 250, respectively. We also included a phenotype file containing EOAD status in the PRSice input and used the software to perform logistic regression (EOAD status ~ LOAD PRS) for each P threshold in each *APOE* model.

#### 4.5.3 Local genetic covariance

Local covariance was calculated using Super GeNetic cOVariance Analyzer (SUPERGNOVA)^76^. LOAD and EOAD summary statistics were prepared by using the munge.py program from LD SCore (LDSC). hg19 formatted summary statistics were used for these steps as they were already prepared including the rsID for each variant as well as this format matched the bfiles and partitioned .bed files provided by the SUPERGNOVA tutorial also in hg19 format.

#### 4.5.4 EOAD and LOAD colocalization

Colocalization between EOAD and LOAD was performed using the coloc.abf function in the Coloc R package. Loci of interest for comparing the two datasets were identified by taking 1MB upstream and downstream of the sentinel SNP in each EOAD GWAS locus. Within each locus, the set of overlapping variants between the two datasets were used to run colocalization. Loci were only considered to colocalize if hypothesis four (variant is causal for both phenotypes) had a posterior probability greater than or equal to 0.8 (PP. ≥ H4 0.8).

### 4.6 Gene Prioritization

To identify likely causal genes within GWAS loci, we employed a summation of scores from a weighted presence-absence matrix for all genes within 1MB flanks of the sentinel variant in GWAS loci. The presence-absence matrix is filled based on evidence of overlap with molecular quantitative loci (QTL), if a gene is significant in gene-based analysis, if a gene is the nearest gene to a significant variant, and if a significant variant is a non-synonymous exonic variant for a gene. Overlap with molecular QTLs was assessed for protein QTLs (pQTL) and expression QTLs (eQTL) in three categories: QTLs for a given gene colocalize with EOAD at a GWAS locus, a given gene has a shared locus with EOAD, or the lead variant in a GWAS locus is a genome-wide significant QTL for a gene. A shared locus is defined as any genome-wide significant QTL within 1MB flanks of the lead variant for a GWAS locus. The specific scoring framework is described by supplementary Table S7.

#### 4.6.1 Generating and accessing QTL data

Cerebrospinal fluid (CSF) pQTL data was generated using CSF levels of 7,584 unique aptamers (6,179 unique proteins) measured on the SOMAscan7k platform for 4,223 participants from six dementia relevant cohorts^30^. pQTL summary statistics used for post-GWAS analyses is generated by meta-analysis of the discovery (n=1,912) and replication (n=1,195) datasets. Cis-pQTLs were variant-aptamer associations within 1MB in either direction of the target protein-coding gene’s hg38 coordinate-based transcription start site. eQTL data were downloaded from GTEx Portal v8 for all Brain tissues, accessed on June 29, 2022. Additional cis-eQTL data was downloaded from the most recent MetaBrain analysis from the MetaBrain website (https://www.metabrain.nl) on downloaded February 24, 2023. CSF metabQTL data was generated using metabolite levels measure by Metabolon for 1,224+1,087 (discovery and replication) participants from five dementia relevant cohorts, while brain metabQTLs are generated using parietal, prefrontal cortex, and temporal cortex post-mortem brain biopsies from 1,172 participants from four dementia-relevant cohorts^54^. CSF metabQTLs analysis is run separately in the discovery and replication sets, then meta-analyzed for the final summary statistics. Further information on pQTL methods can be found in the source material Western et al.,^30^ and metabQTL methods can be found in source material from Wang et al.,^54^.

#### 4.6.2 Colocalization with molecular QTLs

Colocalization with molecular QTLs was performed identically to as described in methods section 4.5.4. All variants within 1MB flanks of the sentinel SNP in each GWAS locus were subset in EOAD summary statistics. QTL data was subset similarly per tissue, per gene. Overlapping variants between the two datasets were used to run colocalization. pQTLs were only considered to colocalize if hypothesis four (variant is causal for both phenotypes) had a posterior probability greater than or equal to 0.8 (PP.H4 ≥ 0.8).

#### 4.6.3 Overlap with molecular QTLs

Overlap with molecular QTLs was queried as described in Methods section 4.6. For each GWAS locus, we determine bounds by taking 1MB upstream and downstream flanks to the lead SNP in the locus. We then subset the molecular QTLs for each gene for each tissue to those loci and query for any genome-wide significant SNPs.

#### 4.6.4 Annotation and gene-based analysis

Annotation and gene-based analyses were performed for each single ancestry and meta-analysis using FUMA software as described in Methods section 4.4. For the purposes of gene prioritization, two annotation items were analyzed. First, a gene was scored if it was the nearest gene to a genome-wide significant variant in the locus. Second, a gene was scored if a genome-wide significant variant in the locus was a non-synonymous exonic (protein altering) variant. For MAGMA gene-based analysis, a gene was scored simply if was significant in gene-based analysis for any ancestry or meta-analysis after multiple testing correction (Bonferroni adjusted P = 0.05 / n genes). The specific cutoff for each ancestry varied, but the number of genes was ~19,000 for each, leading to an approximate cutoff of 2.63×10^−06^.

### 4.7 Biological inference of prioritized genes

Prioritized genes were assessed in four main categories: Primary brain cell type, differential expression in AD brain tissues, association with related phenotypes, biological pathways, and protein-protein interaction. In each we used online methods, protein-based analysis, or pathway analysis, which will be further described below.

#### 4.7.1 Primary Brain cell type

Cell-type specificity analysis was performed by Western et al.,^30^ at the NeuroGenomics and Informatic center at Washington University in St. Louis. The methods are described in full in their publication. As an overview, gene expression data for human astrocytes, neurons, oligodendrocytes, microglia/macrophages, and endothelial cells were downloaded and used to determine a primary cell-type for all SOMAscan 7K panel proteins. Gene expression was averaged across participants for each cell type, then summed for a total expression level for each gene across cell types. Following this, the percentage to which each cell type contributed to the total expression was calculated. A gene was reported to have a specific cell-type if the highest contributing cell type was 1.5× higher than the second highest contributing cell type. For example, if for Gene X, the highest contributing cell type, Cell-type A, was 45% of the total expression, and the next highest cell type, Cell-type B, was 30% or less of the total expression, then Gene X would be Cell-type A specific. Protein in the SOMAscan7K platform were matched to their Entrez gene symbol using the SOMAlogic provided documentation and the ratio-based strategy above was used to determine cell-type specificity for all proteins. EOAD prioritized genes were subset from this to determine cell-type specificity. If cell-type specificity was unable to be determined by this method, the human proteome atlas (https://www.proteinatlas.org/) was also queried to determine if any cell type specificity information was available for a given gene.

#### 4.7.2 Differential Expression in AD brain tissues

The Agora knowledge portal is a database powered by AMP-AD research which hosts evidence for AD relevant genes. The Agora gene comparison tool allows for query and simultaneous comparison of a set of genes’ differential expression as measured by RNA or protein across nine brain regions: anterior cingulate cortex, cerebellum, dorsolateral prefrontal cortex, frontal pole, inferior frontal gyrus, posterior cingulate cortex, parahippocampal gyrus, superior temporal gyrus, and temporal cortex. The tool shows graphically whether any genes in the queried set are significantly upregulated or downregulated in AD diagnosed participants compared to controls. Additionally, the output also provides a genetic score, multi-omics score, and a risk score which sums the multi-omics and genetic score to allow for a numerical assessment of how much a given gene contributes to AD. Agora and AMP-AD methods are further described here https://help.adknowledgeportal.org/apd/AD-Risk-Scores-Data-and-Methods.2826043399.html.

#### 4.7.3 Association with related phenotypes

We used online resources GeneCards^28,29^ (https://www.genecards.org/), the Human Protein Atlas (https://www.proteinatlas.org) and the GWAS catalog^55^ (https://www.ebi.ac.uk/gwas/) to search for our prioritized genes and understand what other phenotypes they are known to affect. Particularly we looked to if they were associated with or known to affect other neurological, dementia and neurodegenerative, cancer, and other health risk factors (e.g., BMI, cardiovascular risk factors, education attainment, etc.).

#### 4.7.4 Biological pathways

Pathway analyses were performed with the enrichGO^78^, enrichDO^79^ DisGeNet and enrichKEGG functions from the R package ClusterProfiler v4.09^80^ as well as the Gene2Func tool from FUMA^72^ and enrichment analyses from STRING^36^. In each case, the input set was all threshold passing genes (Total prioritization score ≥ 4 points) except those from the *APOE* region, and all genes were included as the background.

#### 4.7.5 Protein-protein interactions

Protein-protein interactions were empirically investigated using STRING (https://string-db.org/) database to see if our prioritized genes (excluding the *APOE* region) were enriched for any interactions. Additionally, we performed proteome-wide association (PWAS), which integrates GWAS summary statistics and pQTL data to resolve causal protein in GWAS loci. PWAS was performed with FUSION^81^ transcriptome-wide association study software. Variant weights were calculated for each protein and for each association separately for those aptamers with at least one study-wide pQTL. Protein levels were estimated based on these weights and correlated with EOAD using NHW summary statistic on chromosomes where genome wide significant EOAD GWAS loci were identified. Finally, we performed colocalization between EOAD and trans-pQTLs to see if there was potential evidence of co-regulation of genes in EOAD loci.

## Materials and Methods

1. Cohorts This project used genotype and phenotype data of participants (n = 70,620) who self-identified as either Non-Hispanic White (NHW, n = 50,180), African American (AA, n = 8,563), Asian (n = 4,742), or Hispanic (HIS, n = 2,292) from the Alzheimer’s Disease Genetics Consortium (ADGC) as well as participants from the Knight Alzheimer’s Disease Research Center (Knight-ADRC)(n = 4,843). The ADGC collects data from multiple genotyping rounds from several studies. These include the Adult Changes in Thought (ACT) study, the National Institute on Aging (NIA) Alzheimer’s disease centers (ADC), the Alzheimer’s Disease Neuroimaging Initiative (ADNI), The Predictor of Cognitive Decline Among Normal Individuals study (BIOCARD), the Chicago Health and Aging Project (CHAP), the Children’s Hospital of Philadelphia (CHOP), the Einstein Aging Study (EAS), Glaxo Smith Kline (GSK), the Indianapolis Ibadan Dementia Study (Indianapolis), Johns Hopkins University (JHU), JPN2012, Mayo Clinic Jacksonville (MAYO) and Rochester (RMAYO), The Multi institutional Research of Alzheimer Genetic Epidemiology (MIRAGE) study, the Netherlands Brain Bank (NBB), the National Institute of Aging initiative for Late-Onset Alzheimer’s disease and the National Centralized repository for Alzheimer’s Disease and Related dementias (NIALOAD-NCRAD), the Oregon Health & Science University (OHSU), the Rush University Religious Orders Study/ Memory and Aging Project (ROSMAP), the Texas Alzheimer’s Research and Care Consortium (TARCC), the Translational Genomics Research Institute (TGEN), the University of Miami/Vanderbilt University/Mount Sinai School of Medicine (UMVUMSSM), the University of Pittsburgh (UPITT), and the Washington Heights-Hamilton Heights-Inwood Columbia Aging Project (WHIICAP). Participants from the Knight-ADRC were evaluated by Clinical Core Personnel at Washington University. Cases (CA) were selected on the following bases: a diagnosis of dementia of the Alzheimer’s type, determined by criteria equivalent to the National Institute of Neurological and Communication Disorders and Stroke-Alzheimer’s Disease and Related Disorders Association for probable AD. Severity was evaluated using the Clinical Dementia Rating (CDR). Controls (CO) were assessed by the same criteria and given a nondemented (CDR = 0) diagnosis. Written consent was obtained from all participants. Phenotypic and covariate data from each study was harmonized and merged. For this study, we selected CA with an age of onset (AAO) on the earlier spectrum of AD: AAO 70 or younger. Conversely, CO were selected as cognitively healthy participants who are older than 70 yo at last assessment. Case-Control status for all ADGC participants were clinically defined following ADRC criteria and clinical dementia rating (CDR, must be ≥ 0.5 for cases) guidelines. To create more homogenous groups among participants, we performed principal component analysis (PCA) for all participants. PCA was performed using Plink v1.9^70^ with only very high quality variants (genotyping rate ≥ 99%, MAF ≥ 0.01, and HWE P > 1×10^−06^). Participants were included into analyses for NHW, AA or Asian based on genetic similarity to common genetic ancestries used by HAPMAP. NHW bounds were defined by five standard deviations beyond the means of the first two principal components for trios of Utah residents of northern and western European ancestry (CEU). This was done similarly for AA with respect to Yoruba adult-parent-trios from Ibadan, Nigeria and for Asian with respect to unrelated Japanese individuals from Tokyo, Japan (JPT; Figure S1A). Strict bounds were used to define participants as NHW and Asian since those populations tend to be relatively homogenous. AA and HIS, which are generally more admixed, extend up to the border of NHW and between the borders of NHW and Asian, respectively (Figure S1B). PCA was then performed on each ancestry separately to identify and remove outliers. Finally, to control for cryptic relatedness, we performed Identity by descent (IBD) analysis. Unrelated participants were selected for analysis (Figure S1C) based on pi-hat < 0.198. Within the related pairs, the individual with highest call rate was kept for analysis. After all QC steps, 27,004 (NHW 6,282 CA, 13,386 CO; AA 782 CA, 3,663 CO; Asian 375 CA, 838 CO; HIS 280 CA, 270 CO) participants remained for analysis. Due to low sample size and lack of statistical power, no analyses were performed using the HIS dataset.
2. Genotype QC DNA was genotyped on various arrays, mapped to GRCh38 human genome reference, and imputed using the TOPMed imputation server. The following preparation and QC steps were run on the downloaded genetic data; I) the Variant Call Format (vcf) files were converted to plink using PLINK v1.90b6.26. II) the chromosomal plink files of each study were merged for their respective ancestry. III) variants with R^2^ ≤ 0.3^71^ and variants and participants with genotyping rate (GR) < 98% were removed. IV) variants which were not in Hardy-Weinberg equilibrium (HWE, P < 10^−06^) were removed from autosomal chromosomes. Autosomal and Sex Chromosome data were then merged back into a single plink file. V) Finally, study-specific plink files for each ancestry were merged into a single, ancestry-specific plink file. Genetic data for the Knight-ADRC participants were generated and processed by the Cruchaga Lab at Washington University in St. Louis (https://cruchagalab.wustl.edu/)with identical QC filters as described in Deming et al.,^32^, including a minimum GR 98% and removal of variants from autosomal chromosomes which are not in HWE (P < 10^−06^). After performing QC steps for each ancestry’s phenotype and covariate, we applied a final genotyping rate filter of 90% to maximize the number of high-quality variants as well as minor allele frequency (MAF) filters unique to each dataset based on a minor allele count (MAC) of 5 (NHW MAF ≥ 0.02%; AA MAF ≥ 0.1%, Asian MAF ≥ 0.2%).
3. Statistical Analysis Single variant association analysis was carried out for NHW, AA, and Asian ancestries using plink v2.3. We used a MAC cutoff of five (MAF_NHW_=0.02%, MAF_AA_=0.1%, MAF_Asian_=0.2%), so the number of variants included in each analysis was 12,725,244 for NHW, 19,508,138 for AA and 9,351,864 for Asian. Sex, genotyping array and the first ten principal components (PC) were included as covariates for NHW and AA analyses. Only sex and the first 10 PCs were used for Asian because over 80% of subjects were from a single cohort and genotyped on a single array (Table S2). Age was excluded as a covariate since sample selection is based on age. Additionally, because plink files were merged based on PCA-based ancestry selection, we used a strict HWE filter of P > 1×10^− 30^ in the analysis. For each single variant analysis, significance was set at the standard genome-wide significance threshold of 5×10^− 08^. Following single variant analysis, meta-analysis was performed with a random-effects model using Plink v1.9 “--meta-analysis” function. Variants from meta-analysis used the same significance threshold as single variant analysis. Following initial single variant analysis, stepwise conditional loci was performed at genome-wide significant loci. In stepwise conditional analysis, the lead variant for each locus was included in the model and analysis was re-run on the locus (1MB flanks on the top hit) to identify independent signals. If an independent signal was identified, the new lead variant was included in the model as well and the steps were repeated until there was no longer any significant signal in the locus.
4. Annotation and Gene-based analysis

## Figures and Tables

**Table 1:** Summary of GWAS loci from single variant and meta analyses

			AA		Asian		NHW			
SNP	rsID	NearestGene	BETA	P	Beta	P	BETA	P	Meta P	LOAD_COLOC
**Known Loci**											
chr1:207611623:A:G	rs3818361	*CR1L*	0.070	0.360	−0.041	0.664	0.202	3.20×10^−11^	0.196	0.984*
chr2:127135234:C:T	rs6733839	*BIN1*	0.337	1.07×10^−05^	0.359	0.001	0.221	5.41×10^−18^	1.73×10^−10^	0.999*
chr6:41161514:C:T	rs75932628	*TREM2*	NA	NA	NA	NA	1.274	3.60×10^−16^	3.59×10^−16^	0.999*
chr11:60309220:G:A	rs11824734	*MS4A6E*	−0.027	0.740	−0.014	0.908	−0.154	2.55×10^−09^	0.033	0.787*
chr11:86109035:A:G	rs543293	*PICALM*	−0.185	0.079	−0.107	0.254	−0.153	1.03×10^−08^	1.28×10^−09^	0.995*
chr19:44893408:G:T	rs59007384	*T0MM40*	0.261	0.002	0.108	0.009	1.160	1.25×10^−351^	0.088	1.00**
chr19:54302959:T:C	rs2781753	*LILRA5*	−0.014	0.853	NA	NA	−0.167	1.96×10^−11^	0.150	0.059*
**Novel Loci**											
chr2:132116109:ATG:A	rs202077887	*ANKRD30BL*	0.052	0.524	−0.204	0.446	0.683	4.88×10^−15^	0.427	0.028*
chr3:73046958:A:C	rs138671104	*EBLN2*	−0.131	0.820	NA	NA	0.634	1.67×10^−10^	0.152	0.018*
chr5:21360122:T:C	rs55965129	*GUSBP1*	0.015	0.877	−0.229	0.108	0.350	3.57×10^−09^	0.708	0.013*
chr11:49032935:G:A	rs11040229	*TRIM49B*	0.160	0.058	0.126	0.351	0.170	9.53×10^−08^	1.02×10^−08^	0.059*
chr11:50142106:G:A	rs111535109	*AC109635.7*	−0.081	0.695	0.227	0.257	−0.428	9.33×10^−13^	0.559	0.04*
chr12:37412586:T:G	rs1349119633	*AK6P2*	−0.019	0.877	NA	NA	0.452	6.70×10^−10^	0.335	0.043**
chr19:18422832:T:C	rs7258465	*SSBP4*	0.180	0.021	0.130	0.218	0.139	1.30×10^−07^	4.44×10^−09^	0.102**
chr21:13917037:C:T	rs4432555	*ANKRD20A11P*	−0.168	0.271	0.036	0.764	0.243	8.39×10^−10^	0.572	0.011*

This table summarizes the nearest gene, the prioritized gene, and summary statistics from each ancestry’s single variant analysis, meta-analysis and LOAD GWAS locus. Known AD loci are shown at the top of the table, novel loci are in the lower half of the table. LOAD_COLOC is the best PPH4 value from doing colocalization between EOAD and LOAD at EOAD loci, while LOAD_P is the best p value for the lead SNP in LOAD analyses.

* = best is from Bellenguez et al., from Shigemizu et al.,

*** = best is from Kunkle et al.,

## Abbreviations

AA: African American
AAO: age at onset
ACC: Anterior cingulate cortex
ACT: Adult changes in thought
ADC: Alzheimer’s disease centers
ADGC: Alzheimer’s Disease Genetics Consortium
ADGC: Alzheimer’s Disease
ADGC: Alzheimer Disease
ADNI: alzheimer’s disease neuroimaging initiative
Aging: national institute on Aging
Aβ: amyloid beta
BIOCARD: predictor of cognitive decline among normal individuals
BP: biological processes
CA: Case/Cases
CBE: cerebellum
CC: cellular components
CDR: clinical dementia rating
CHAP: chicago health and aging project
CHOP: children’s hospital of philidelphia
Chr: chromosome
CO: Control/Controls
CSF: cerebrospinal fluid
DEG: differentially expressed gene/genes
DGN: disease–gene network
DO: disease ontology
EAS: Einstein aging study
EOAD: Earlier–onset Alzheimer disease
eQTL: expression quantitative trait loci/locus
ER: endoplasmic reticulum
FACE: Fundacio ACE
FUMA: Functional Mapping and Annotation
GO: gene ontology
GR: genotyping rate
GSK: Glaxo smith kline
GWAS: genome–wide association study
HIS: Hispanic
HWE: hardy–weinberg equilibrium
IBD: identity by descent
IFG: inferior frontal gyrus: indianapolis: indianapolis ibadan dementia study
JHU: Johns hopkins university
Knight: ADRC–Charles F. and Joanne Knight Alzheimer’s Disease Research Center
L2FC: log2–fold change
LD: Linkage disequilibrium
LDSC: Linkage disequilibrium score regression
LOAD: Late–onset Alzheimer disease
MAC: minor allele count
MAF: minor allele frequency
MAGMA: Multi–marker analysis of genomic annotation
MAYO: Mayo clinic jacksonville: metabQTL: metabolite quantitative trait loci/locus
MF: molecular functions
MIRAGE: Multi–institutional research of Alzheimers genetic epidemiology
N/n: sample size/number
NBB: netherlands brain bank
NCA: number of cases
NCO: number of controls
NHW: non–Hispanic White
NIALOAD: NCRAD–national institute of aging initiative for Late–onset Alzheimer’s disease and the national centralized repository for Alzheimer’s disease and related dementias
OHSU: Oregon Health and Science university
PC: principal component
PCA: principal component analysis
PHG: parahippocampal gyrus
PPH4: posterior probability for hypothesis 4
PPI: protein–protein interaction
pQTL: protein quantitative trait loci/locus
PRS: polygenic risk score
PWAS: proteome–wide association study
QTL: quantitative trait loci/locus
rg: genetic correlation
RMAYO: mayo clinic rocherster
ROSMAP: rush university religious orders study/memory and againg project
SNP: single nucleotide polymorphism
STG: superior temporal gyrus
SUPERGNOVA: Super genetic covariance analyzer
TARCC: texas alzheimer’s research and care consortium
TGEN: translational genomics research institute
TWAS: Transcriptome–wide association study
UMVUMSSM: the university of Miami/Vanderbilt university/Mount Sinai school of medicine
UPITT: university of Pittsburgh
vcf: variant call format
WASHU: Washington University in St. Louis
WHIICAP: Washington heights–Hamilton heights–inwood columbia aging project
yo: years old
